# Decreased Mutation Frequencies among Immunoglobulin G Variable Region Genes during Viremic HIV-1 Infection

**DOI:** 10.1371/journal.pone.0081913

**Published:** 2014-01-07

**Authors:** Elisabeth Bowers, Ronald W. Scamurra, Anil Asrani, Lydie Beniguel, Samantha MaWhinney, Kathryne M. Keays, Joseph R. Thurn, Edward N. Janoff

**Affiliations:** 1 Mucosal and Vaccine Research Program Colorado (MAVRC), University of Colorado Denver, Aurora, Colorado, United States of America; 2 Infectious Disease Division, University of Colorado Denver, Aurora, Colorado, United States of America; 3 Department of Microbiology, University of Colorado Denver, Aurora, Colorado, United States of America; 4 Colorado School of Public Health, University of Colorado Denver, Aurora, Colorado, United States of America; 5 Denver Veterans Affairs Medical Center, Denver, Colorado, United States of America; 6 Minneapolis VA Health Care System, University of Minnesota, Minneapolis, Minnesota, United States of America; 7 GIMAP EA 3064, Faculté de Médecine, Université Jean Monnet, Saint Etienne, France; South Texas Veterans Health Care System and University Health Science Center San Antonio, United States of America

## Abstract

**Background/objective:**

HIV-1 infection is complicated by high rates of opportunistic infections against which specific antibodies contribute to immune defense. Antibody function depends on somatic hypermutation (SHM) of variable regions of immunoglobulin heavy chain genes (V_H_-D-J). We characterized the frequency of SHM in expressed IgG mRNA immunoglobulin transcripts from control and HIV-1-infected patients.

**Design:**

We compared utilization of genes in the most prominent V_H_ family (V_H_3) and mutation frequencies and patterns of cDNA from V_H_3-IgG genes from 10 seronegative control subjects and 21 patients with HIV-1 infection (6 without and 15 patients with detectable plasma viremia).

**Methods:**

Unique IgG V_H_3 family cDNA sequences (n = 1,565) were PCR amplified, cloned, and sequenced from blood. Sequences were analyzed using online (Vbase) and in-house immunoglobulin alignment resources.

**Results:**

Mutation frequencies in the antigen-binding hypervariable complementarity determining regions (CDR1/2) of IgG class-switched B cells were lower among viremic HIV-1-infected patients vs. controls for nucleotides (CDR1/2: 10±5% vs. 13.5±6%, p = 0.03) and amino acids (CDR: 20%±10 vs. 25%±12, p = 0.02) and in structural framework regions. Mutation patterns were similar among groups. The most common V_H_3 gene, V_H_3-23, was utilized less frequently among viremic HIV-1-infected patients (p = 0.03), and overall, mutation frequencies were decreased in nearly all V_H_3 genes compared with controls.

**Conclusions:**

B cells from HIV-1-infected patients show decreased mutation frequencies, especially in antigen-binding V_H_3 CDR genes, and selective defects in gene utilization. Similar mutation patterns suggest defects in the quantity, but not quality, of mutator activity. Lower levels of SHM in IgG class-switched B cells from HIV-1-infected patients may contribute to the increased risk of opportunistic infections and impaired humoral responses to preventative vaccines.

## Introduction

B cell activation and hypergammaglobulinemia are among the first and most persistent immunologic consequences of HIV-1 infection [1]–[2]. High rates of infection and impaired humoral responses to vaccines during HIV-1 infection may be related to an impaired ability to generate pathogen-specific antibodies in sufficient quantities, but also of sufficient quality and function to control these pathogens [3]–[5]. The successful evolution of antibody diversity, specificity and function is determined by three distinct processes. First, antigen-independent recombination of variable (V), diversity (D) and joining (J) gene segments establishes the primary repertoire in naïve B cells (IgD^+^IgM^+^) and appears relatively intact during HIV-1 infection [6]. Subsequently, in lymph node germinal centers, antigen-dependent somatic hypermutation (SHM) modifies the antigen-binding variable regions of the heavy (V_H_) and light (V_L_) chains, which, following selection, enhances antigen specificity and avidity [7]. Finally, class-switch recombination (CSR) modifies the effector constant regions of the heavy chain (C_H_) to a single isotype (IgG, IgA or IgM) and may be somewhat impaired during HIV-1 infection [8]–[9].

We focused on class-switched IgG sequences of the largest of the 7 immunoglobulin V_H_ gene families, V_H_3. The V_H_3 family comprises 22 of 44 functional human V_H_ genes [10] and encodes most antibodies to capsular polysaccharides of common HIV-1-associated pathogens (e.g. *Streptococcus pneumoniae*, *Haemophilus influenzae*, *Cryptococcus neoformans*, and *Salmonella* spp.) [11]–[13]. We show that, compared with uninfected control subjects, viremic HIV-1 infection is associated with significantly decreased frequencies of SHM in CDR1/2 (nucleotides and amino acids) of V_H_3 genes. Because antibody avidity and function are determined by SHM, these decrements in V_H_3 mutation may contribute to the increased rates of primary and recurrent infections against which antibodies contribute to protection, and to the limited efficacy of polysaccharide vaccines to protect against these pathogens in this adult population [14]. The mechanisms of HIV-1-associated defects may include decreases in the frequency or magnitude but not the quality of the SHM process mediated by activation-induced deaminase (AID) protein, related DNA repair enzymes, or antibody selection in germinal centers.

## Methods

### Population Studied

We enrolled 31 adults, including10 HIV-1-seronegative control subjects with no known risks for HIV-1 infection and 21 patients with HIV-1 infection and <400 CD4^+^ T cells/ul: 6 on antiretroviral therapy with no detectable plasma HIV-1 RNA (HIV+ Aviremic) for >6 months and 15 with detectable plasma HIV-1 RNA (HIV+ Viremic) with or without therapy (Table 1). Exclusion criteria included acute medical illness, underlying organ dysfunction (e.g., renal, hepatic, cardiac) or immunosuppressive therapy and for control subjects, any risks for HIV-1 infection. Written informed consent was obtained with protocols approved by Institutional Review Boards at Veterans Affairs Medical Centers in Minneapolis and Denver and the Universities of Minnesota and Colorado Denver.

**Table 1:** Clinical Characteristics of Study Subjects.

	Control	Aviremic	Viremic	p value
**Number**	10	6	15	
**Age – median years (range)**	28 (24–52)	53.5 (44–59)	43 (26–52)	0.001^a^
**Sex (M∶F)** b	6∶4	6∶0	14∶1	0.04^c^
**% Non-Caucasian** d	0%	33%	33.3%	0.06^e^
**CD4^+^ T cells/uL – median (range)**	N/A	270 (129–316)	140 (34–394)	0.05
**HIV-1 RNA – median copies/mL (range)**	N/A	<50	56,324 (500–1×10^6^)	
**Antiretroviral Therapy** f				
**-No Therapy**	N/A	0	10 (67%)	
**-1 medication**	N/A	0	2 (13%)	
**-≥3 medications**	N/A	6 (100%)	3 (20%)	
**Serum Immunoglobulins** g				
**IgG – median mg/dL (range)**	1212 (837–1541)	1445 (1110–2390)	2070 (773–4630)	0.003^h^
**IgA – median mg/dL (range)**	191 (120–412)	297 (174–397)	333 (27–1090)	0.25
**IgM – median mg/dL (range)**	132 (92–288)	112 (57–232)	222 (48–682)	0.02^i^
**Spontaneous ex vivo Ig production** j				
**IgG – median ng/mL (range)**	55 (44–143)	256 (73–1094)	532 (33–12441)	0.04^k^
**IgA – median ng/mL (range)**	26 (2–70)	269 (88–1397)	191 (35–8093)	0.01^l^
**IgM – median ng/mL (range)**	11 (2–24)	2 (2–106)	36 (2–153)	0.14

Overall p values are listed on the table.

^a^ Secondary analysis: control vs. aviremic p<0.0001, all other comparisons p>0.05.

^b^ Effect of gender was evaluated by Chi Square test.

^c^ Secondary analyses were calculated by Fisher's exact test: control vs. aviremic p = 0.23, control vs. viremic p = 0.12, aviremic vs. viremic p = 1.00.

^d^ Non-Caucasian = African-American, Asian/Pacific Islander, Native American, Hispanic, or Other; overall p value calculated by Chi Square test.

^e^ Secondary analyses were calculated by Fisher's exact test: control vs. aviremic p = 0.04, control vs. viremic p = 0.06, aviremic vs. viremic p = 0.63.

^f^ Antiretroviral therapy: 1 medication = NRTI, ≥3 medications = 1 PI+2 NRTI or NNRTI+2 NRTI.

^g^ Control n = 9, aviremic n = 6, viremic n = 15.

^h^ Secondary analyses: control vs. viremic 0.01<p<0.05, all other comparisons p>0.05.

^i^ Secondary analyses: aviremic vs. viremic p<0.05, all other comparisons p>0.05.

^j^ Immunoglobulins were measured in supernatants from 10^5^ unstimulated PBMC cultured in RPMI+10% heat-inactivated fetal calf serum for 7 days, control n = 5, aviremic n = 6, viremic n = 7.

^k^ Secondary analyses: control vs. viremic p<0.05, all other comparisons p>0.05.

^l^ Secondary analyses: control vs. aviremic p<0.05, control vs. viremic p<0.05, aviremic vs. viremic p>0.05.

### Total Immunoglobulins

Total IgG, IgM and IgA in sera were measured by nephelometry, and in supernatants from PBMC (see below) by enzyme immunoassay [8].

### Peripheral Blood Mononuclear Cells (pbmc)

PBMC were separated from 60 mL of fresh whole blood by density centrifugation. For total immunoglobulin production, unstimulated cells (1×10^5^/well in triplicate) were cultured in RPMI (Gibco, Carlsbad, CA) with L-glutamine and 10% heat-inactivated fetal calf serum for 7 days. For RNA, 5–10 million cells were suspended in Trizol (Invitrogen) or RNALater (Applied Biosystems Inc., Foster City, CA) and stored at −20°C. RNA was extracted using Trizol or an RNEasy RNA Extraction Kit (Qiagen, Inc., Valencia, CA), quantified using a NanoDrop spectrophotometer (NanoDrop Technologies Inc., Wilmington, DE), and stored at −80°C. First strand cDNA synthesis was performed as described [6].

### Pcr Amplification Of V_H_3 Igg Genes

Primers used for PCR amplification were commercially synthesized primers derived from the IgG constant gene (3′IgG: 5′-GGGTGCCAGGGGGAAGACCG-3′; or CH2A: 5′-CACCGGTTCGGGGAAGTAGTCC-3′ and SC-CH2A: 5′-CCTTCGCCGACTGACACCGGTTCGGGGAAGTAGTCC-3′) and the V_H_3-family specific leader primer (5′VH3: 5′-CCATGGAGTTGGGGCTGAGCTGC-3′ [15]–[17]). For cloning using a TOPO TA kit, PCR (20 ul) consisted of: 0.5 ul of cDNA, 4 nmol each dNTP, 40 nmol MgSO_4_, 2 ul of 10× High Fidelity PCR buffer, 4 pmol each primer (3′IgG and 5′VH3), 0.1 ul Platinum® Taq High Fidelity DNA polymerase (Invitrogen). Samples were heated to 94°C for 30 seconds in a Perkin-Elmer 9700 thermocycler (“hot start”), followed by 24 cycles consisting of 30 seconds of 94°C denaturation, 30 seconds of 60°C annealing, and 30 seconds of 68°C extension. For cloning using a StabyCloning kit, PCR (25 ul) consisted of: 1 ul cDNA, 7.5 nmol each dNTP, 25 nmol MgSO_4_, 2.5 ul 10× Pfx Amplification buffer, 3 pmol CH2A primer (10 uM), 6 pmol 5′VH3 primer, 7.5 pmol SC-CH2A primer, 0.2 ul Platinum Pfx DNA polymerase (Invitrogen). Samples were heated to 94°C for 2 minutes in an Applied Biosystems 9700 thermocycler (“hot start”), followed by 45 cycles consisting of 30 seconds of 94°C denaturation, 30 seconds of 60°C annealing, and 30 seconds of 68°C extension. PCR product was purified using a Qiagen QIAquick PCR Purification kit (Qiagen).

### Determination Of Polymerase Fidelity

Comparison of an 18 bp segment of IgG constant regions from 823 IgG sequences with germline sequences yielded a maximum potential error rate of 1.15×10^−3^ (17 mutations/14,814 bp), or ∼1 error/871 bp (or ∼1 error per 2.5 V_H_ genes). Thus, only ∼1.7% of the mean of 20 measured mutations/V_H_3 gene in controls could be ascribed to polymerase amplification errors.

### Cloning And Sequencing Of V_H_3 Igg Clones

PCR products (1–4 uL) were cloned into a pCR®-4TOPO vector using a TOPO TA Cloning® kit (Invitrogen) or pSTC1.3 vector (10 ul of PCR product) using a StabyCloning kit (Eurogentec, San Diego, CA). Plasmids from bacterial colonies cultured overnight in LB medium (100 ug/ml ampicillin) were purified using a QIAprep® Spin Miniprep kit (Qiagen). Plasmid inserts were sequenced using SequiTherm Excell (Epicentre Technologies, Madison, WI), as described [6], or by the Colorado Cancer Center Core Sequencing Facility using an ABI3730 Sequencer (Applied Biosystems, Foster City, CA). Sequences are accessible in the GenBank database, www.ncbi.nlm.nih.gov/genbank.

### V_H_3 Sequence Analysis

Somatic hypermutations were identified by comparing the sequences to the Vbase (http://vbase.mrc-cpe.cam.ac.uk/) database of variable region germline sequences using the DNAplot software. Multiple identical sequences were excluded from analysis and the percent of sequences excluded did not differ between group (median percent of redundant sequences relative to the total number of sequences per patient; control = 24.2%, aviremic = 31.5%, viremic = 21.1%, overall p = 0.70). Conservative amino acid substitutions in either direction included: Ala to Gly, Ile, or Leu; Ile to Leu, Met, Phe, or Val; Leu to Met, Phe, or Val; Arg to His or Lys; Gly to Ile, Leu, or Val; Asp to Glu; Asn to Gln; His to Lys; Met to Phe or Val; Phe to Val; and Ser to Thr. All other substitutions which showed changes in charge or polarity were considered non-conservative. Three nucleotide/amino acid insertions and deletions within sequences relative to the reference sequence all fell within the CDR1/2 regions and were not included in analyses. The number of these did not differ between group (median percent of insertion/deletions relative to total number of amino acids in the sequence; control = 0.03%, aviremic = 0%, viremic = 0.02%, overall p = 0.12). D regions were identified using the criteria of a minimum of 6 bp identity over a 7 bp length [18].

### Analysis Of V_H_3 Cdr3 Regions

The first amino acid in CDR3 was assigned as the third amino acid following a conserved cysteine residue at position 92 (Kabat numbering) of the V_H_ gene [15]. The last amino acid was assigned just before the first conserved tryptophan of FR4 (Kabat position 103). The CDR3 sequences were analyzed for length, composition of residues, and average hydrophobicity using the Kyte-Doolittle scale [19] as normalized by Eisenberg [20] using a Batch Analyzer program developed and validated in our group by comparison with online programs (Vbase, IMGT® [21] and JOINSOLVER® [22]).

### Statistics

Analyses used two-sided tests with a significance level of 0.05. Differences in the overall (primary) comparison of percent medians between the groups were analyzed using a nonparametric Wilcoxon rank sum test. A Fisher's projected least significant different approach was utilized for secondary analyses, such that pairwise comparisons are conducted only if the overall test was significant. For each subject, the expression of specific V_H_3 family genes was compiled and the mean percent expression was calculated; the median percent expression was calculated from the means of the individual patients for each group. Generalized linear models (binomial family) were used for comparing the proportion of events between groups.

## Results

### Immunoglobulin Production

Consistent with the earliest descriptions of HIV-1 infection, levels of total IgG were significantly increased in viremic HIV-1-infected patients compared with controls (Table 1). Levels of IgG in successfully-treated patients without detectable viremia (aviremic) were comparable to those in seronegative control subjects. Similarly, in culture, IgG and IgA produced spontaneously by PBMC over 7 days were significantly higher among viremic HIV-1-infected patients. Despite differences in quantity, the proportions of each isotype produced *in vitro* (IgG 51.5–63.7%; IgA 28.3–45.8%; IgM 2.8–12%) and measured in serum *in vivo* (IgG 74.2–77.8%; IgA 13.3–17%; IgM 6.1–9.8%) were comparable between groups. Thus, the ability of B cells to class switch from IgM to IgG or IgA in the absence of specific antigenic stimuli appears intact in our HIV-1-infected cohort.

### V_H_3 Gene Expression

**Figure 1:**
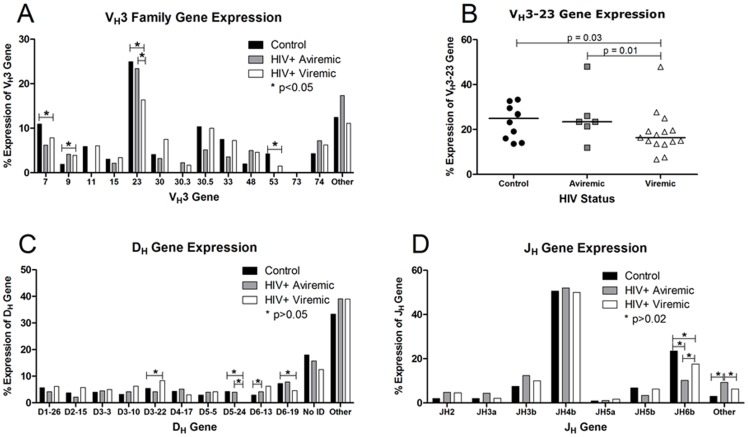
Expression of V_H_3, D, and J family genes. Data are shown as group medians among HIV-1-seronegative control subjects (n = 10; black bars or circles), aviremic patients (n = 6; gray bars or squares), and viremic HIV-1-infected patients with detectable plasma HIV-1 RNA (n = 15; white bars or triangles). Values were calculated from individual mean percent expression for each gene. A) V_H_3 family gene expression. “Other” includes V_H_3-13, -20, -43, -49, -64, -65, -66, -72, and unidentified genes which ranged from 0.4% to 12.6% expression. P values were calculated by Mann-Whitney test. Significant p values were found for V_H_3-7 (p = 0.05), V_H_3-9 (p = 0.05), V_H_3-23 (p = 0.03), and V_H_3-53 (p = 0.007). B) V_H_3-23 expression. Each point represents the mean percentage of V_H_3-23/total V_H_3 genes for each patient. The solid bar indicates the group median. C) D gene expression was calculated as with V_H_3 gene expression. “Other” includes D1-1, D1-7, D1-14, D1-20, D2-2, D2-8, D2-21, D3, D3-9, D3-16, D4, D4-b, D4-11, D4-23, D5-12, D6-6, and D6-25 which ranged from 0.4% to 4.3% expression. “No ID” (12.5–18.0% of sequences) indicates that gene identification could not be assigned. P values were calculated by Mann-Whitney test. Significant overall p values were found for D3-22 (p = 0.04), D5-24 (p = 0.05), and D6-19 (p = 0.04). D) J gene percent expression was calculated as described above. “Other” includes JH1, JH4a, JH4d, JH6a, and JH6c which ranged from 0.2% to 6.7% expression. Statistics were also calculated as above. A significant overall p value was found for JH6b (p = 0.01).

V-D-J gene recombination is the first antigen-independent step in generating the antibody repertoire. We characterized V_H_3-gene utilization by cloning and sequencing 494 IgG-V_H_3 cDNA clones from circulating class-switched B cells from 10 control subjects, 793 clones from 15 HIV-1-infected patients with HIV-1 viremia (80%>10,000 copies/mL) and 278 clones from 6 HIV-1-infected aviremic patients (median 50 clones/subject; range 40–66; accession numbers JN576421–JN577983). Of the 22 individual V_H_3 genes, at least 20 were expressed in each group. V_H_3-23, although the most frequent (Figure 1a), was also significantly decreased among viremic HIV-1-infected patients compared with controls (Figure 1b), as were V_H_3-7 and V_H_3-53 (Figure 1a). This decrease was consistent for most viremic patients, among whom the frequency of V_H_3-23 expression was below the median for control subjects in 13 of 15 patients (Figure 1b).

Only limited differences were observed in the utilization of the other variable region genes, D and J. D regions, located centrally in the hypervariable CDR3 region and most commonly mutated, could be assigned in 84.6% of sequences. We identified few significant differences in utilization of less prevalent D genes (Figure 1c). Similarly, utilization of J_H_ segments was comparable in each group, with J_H_4b comprising half of all J_H_ segments (Figure 1d). Thus, as suggested in earlier work on naïve B cells [6], the repertoire of expressed variable region genes appears fundamentally intact in patients with HIV-1 infection.

### Cdr3 Characteristics

The CDR3 region typically plays the greatest role in antigen binding [23] and has the greatest degree of diversity. The length of the CDR3 region, which overlaps the junctions of the V_H_, D, and J_H_ genes, was not significantly different between all groups (Table 2). Hydrophobicity, resulting from hydrophobic residues buried within the protein, was also comparable between groups, as was CDR3 amino acid composition.

**Table 2:** Biochemical characteristics of amino acids in the CDR3 regions in HIV-1-infected and control subjects.

	Control	Aviremic	Viremic	p value
**CDR3 Length (amino acids)**	13.1 (12.2–14.2)	12.7 (11.1–12.9)	13.4 (11.5–15.1)	0.02^a^
**Hydrophobicity Index**	−0.009 (−0.082–0.047)	−0.0022 (−0.05–0.02)	0.0014 (−0.072–0.096)	0.66
**Acidic Residues**	15.9% (14.6–19.2)	17.5% (14.3–18.7%)	15.7 (13.0–18.2)	0.30
**Basic Residues**	7.5 (6.2–9.9)	6.7% (6.1–8.7%)	7.3 (5.6–9.2)	0.57
**Uncharged Polar Residues**	42.7 (36.1–46.7)	42.4% (37.2–43.8%)	43.6 (35.9–47.6)	0.63
**Nonpolar Residues**	33.4 (29.9–36.5)	33.4 (31.0–37.6%)	34.1 (29.7–40.1)	0.91
**Aromatic Residues**	24.9 (18.1–29.0)	23.8% (21.6–27.7%)	24.3 (22.9–27.7)	0.53

CDR3 length and Hydrophobicity Index were calculated as described in the methods. Residue characteristics were calculated with the number of each type of residue divided by the total number of residues in the CDR3 region of each sequence. Values are listed as the group median with the range of individual mean percents in parentheses. Overall p values are listed on the table.

^a^ Secondary analyses: aviremic vs. viremic p<0.05; all other comparisons p>0.05.

### Reduced Mutation Frequency In Hiv-1-Infected Patients

**Figure 2:**
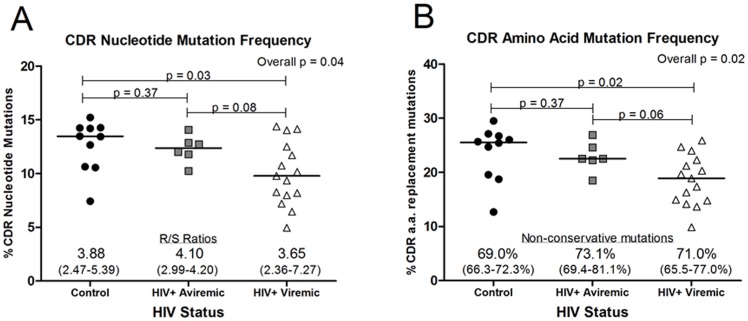
Mutation frequency is reduced in CDR1/2 regions of V_H_3 genes from viremic HIV-1-infected patients. The mean percent of mutated nucleotides or the mean percent of replaced amino acids was calculated for each control subject (n = 10; black circles), aviremic patient (n = 6; gray squares), or viremic HIV-1-infected patient (n = 15; white triangles) based on the alignment of the cloned sequences with V_H_3 sequences from the Vbase immunoglobulin database. The solid bar indicates the group median. A) The mean percent of mutated nucleotides in the CDR1/2 regions. The median (and range) of replacement to silent (R/S) ratios indicates the number of amino acids replaced as a result of nucleotide mutations relative to unchanged (silent) amino acids (overall p = 0.84). B) The mean percent of replaced amino acids in the CDR1/2 regions. The median (and range) percent non-conservative mutations was calculated relative to the total number of replaced amino acid mutations (overall p = 0.02, secondary analyses: control vs. aviremic p<0.05, all other comparisons p>0.05).

SHM is typically an antigen-driven process that enhances antibody affinity and function. Hypervariable complementarity determining regions (CDR), which are the principal antigen-binding regions and the primary targets for SHM, were assessed. The nucleotide mutation frequency in the CDR1/2 regions of IgG-V_H_3 sequences from the viremic group was significantly lower than that of controls (p = 0.03; Figure 2a), whereas values for aviremic patients on effective antiretroviral therapy were not different from controls. The ratio of replacement to silent (R/S) amino acid changes resulting from these nucleotide mutations, one indicator of positive antigen-driven selection, was high and similar in each group. Although the frequency of predicted amino acid mutation in CDR1/2 was lower among the viremic patients compared with controls (Figure 2b), over two-thirds of the amino acid mutations were non-conservative in each group, again consistent with antigen selection.

**Figure 3:**
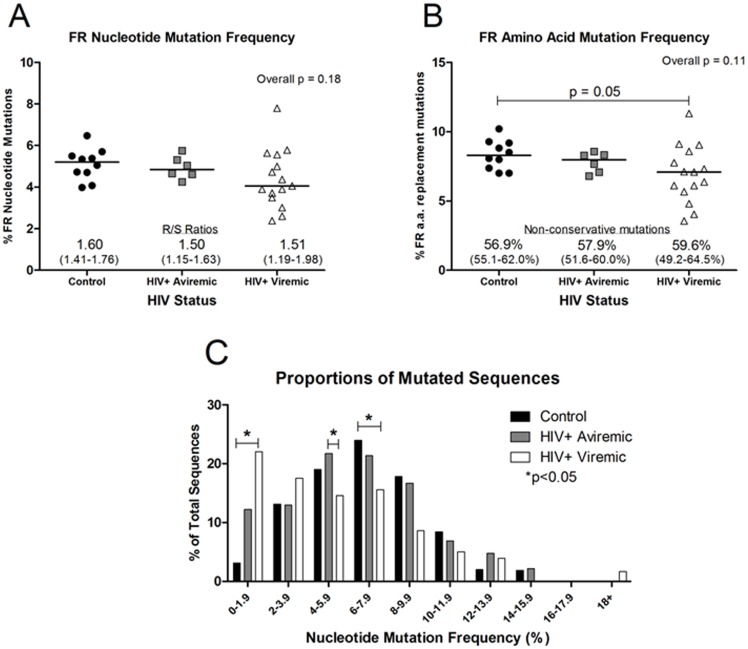
Amino acid mutation frequency is reduced in FR1/2/3 regions of viremic HIV-1-infected patients. The mean percent of mutated nucleotides or the mean percent of replaced amino acids was calculated for each control subject (n = 10; black circles), aviremic patient (n = 6; gray squares), or viremic HIV-1-infected patient (n = 15; white triangles) based on the alignment of the cloned sequences with V_H_3 sequences from the Vbase immunoglobulin database. The solid bar indicates the group median. A) The mean percent of mutated nucleotides in the FR1/2/3 regions. The median (and range) of R/S ratios indicates the number of amino acids replaced as a result of nucleotide mutations relative to unchanged (silent) amino acids (overall p = 0.17). B) The mean percent of replaced amino acids in the FR1/2/3 regions. The median (and range) percent non-conservative mutations was calculated relative to the total number of replaced amino acid mutations (overall p = 0.19). C) The proportion of sequences with low nucleotide mutation density is greater in viremic HIV-1-infected patients. The median proportion of sequences with specific nucleotide mutation density across the entire sequence is graphed by group (control = black bars, aviremic = gray bars, viremic = white bars). Overall p values 0–1.9%, p = 0.02; 4–5.9%, p = 0.04; 6–7.9%, p = 0.04. Secondary analyses are shown on the graph. Proportions are expressed as a percentage of the total number of sequences.

For the structural framework (FR1/2/3) regions, the nucleotide mutation frequency, as expected [24], was lower than in CDR1/2 regions (2–8% vs. 5–15%, respectively; Figures 3a vs. 2a). The variance in FR nucleotide and amino acid mutation frequencies was greater in viremic patients than in either aviremic patients or controls, but significantly different only for amino acids (Figure 3a, 3b). The R/S ratios were also lower in FRs than in CDRs but similar between groups. The frequency of non-conservative replacements was lower in FR amino acids (3–12%, Figure 3b) compared with CDRs (10–30%, Figure 2b), but did not vary between groups. Results from aviremic patients were most similar to those of control subjects.

The mutation frequencies were lower in sequences from viremic patients compared with controls, but the median proportions of unmutated sequences did not differ between any group (control: median = 1.14%, range = 0–12.8%; aviremic: median = 1.07%, range 0–4.3%; viremic: median = 7.69%, range = 0–18.8%; overall p = 0.26), suggesting that the lower frequencies were not due to skewing by IgG class-switched but unmutated sequences, as have been described in young infants [25]. Rather, the decreased density of nucleotide mutations per sequence (whole sequence) in the viremic group was distributed throughout the antibody population (Figure 3c). Neither the frequencies of mutations of nucleotides nor amino acids in CDR1/2 or FR1/2/3 correlated significantly with either plasma HIV-1 RNA levels (r = 0.46, p = 0.30) or CD4^+^ T cell numbers (r = 0.15, p = 0.63, data not shown).

### V_H_3 Gene-Specific Shm

**Figure 4:**
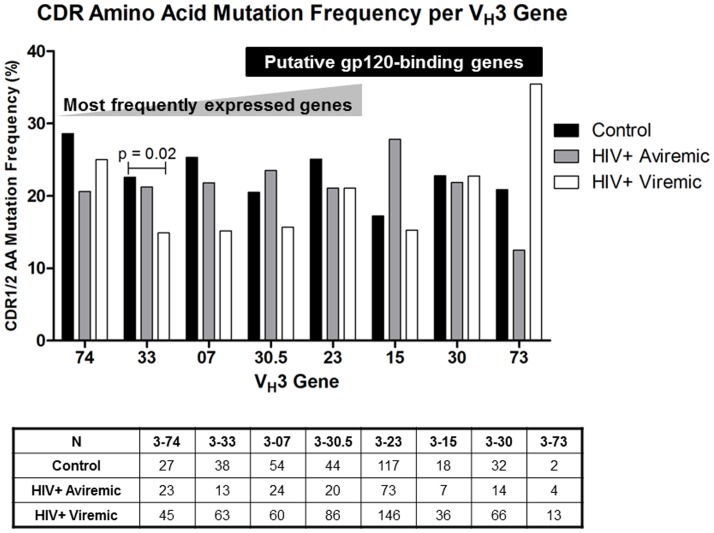
Frequencies of amino acid replacement mutations in specific V_H_3 genes. The group median for CDR1/2 in each of the 5 most frequently expressed V_H_3 genes (V_H_3-74, -33, -07, -30.5, and -23) and for the V_H_3 genes proposed to bind to HIV-1 gp120 outside of the antigen binding region (V_H_3-30.5, -23, -15, -30, and -73) are calculated from the mean mutation frequencies for each individual subject in control (n = 10; black bars), aviremic (n = 6; gray bars), and viremic groups (n = 15; white bars). The table below shows the total number of sequences cloned (N) for each gene by group. P values were calculated for each gene. Only the overall p value for V_H_3-33 was significant (p = 0.02). Secondary analyses: control vs. viremic p<0.05; all other comparisons p>0.05.

The decrement in mutation frequency in CDR1/2 of viremic patients was present across nearly all V_H_3 family genes, including the 5 most commonly expressed genes, representing 53% of all sequences analyzed (833/1,565) (Figure 4). Differences were statistically significant only for V_H_3-33 (overall p = 0.02). Products of 5 V_H_ genes (V_H_3-30.5, -23, -15, -30, and -73) have been proposed to bind the HIV-1 envelope gp120 in a nontraditional manner outside the antigen-binding pocket [26], potentially as a superantigen-like stimulus for selective deletion of B cells. However, we found no consistent significant differences in the pattern of amino acid replacement frequencies for any of these genes between any groups.

### Topographical Pattern Of Amino Acid Mutations

**Figure 5:**
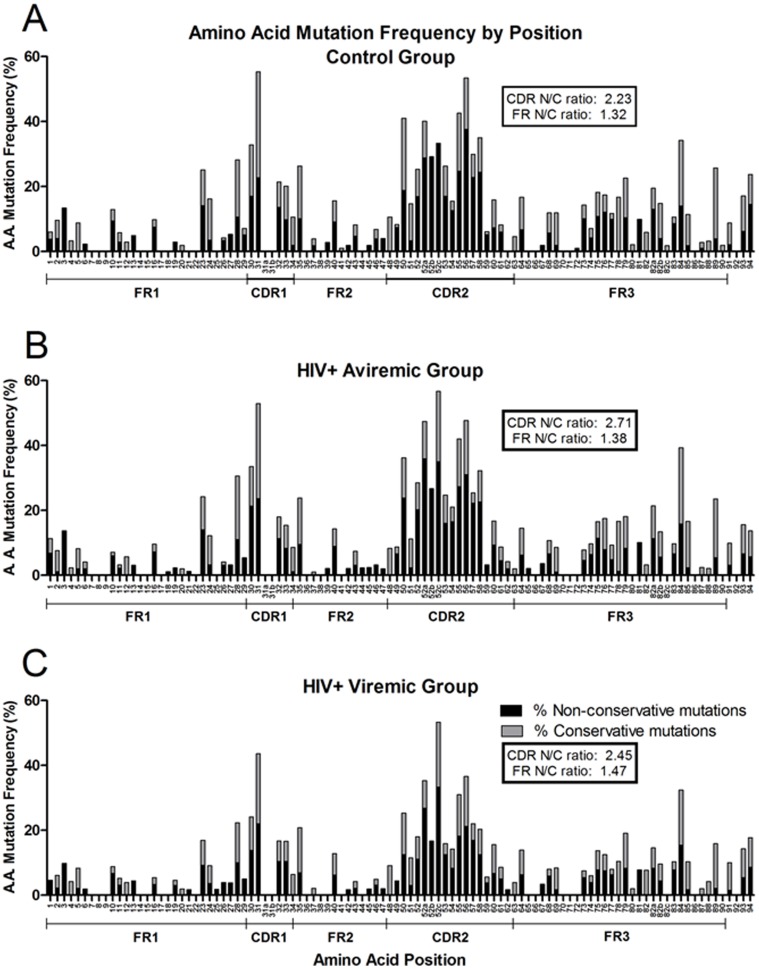
Amino acid mutation frequencies at each position do not differ significantly among groups. The percent of non-conservative (N; black bars) or conservative (C; gray bars) mutations relative to the total number of amino acids sampled at each position (Kabat numbering) are represented, the sums of which equal the total percent replaced amino acid mutation frequency at each position. The mean percent replacement amino acid mutation frequency was calculated for each individual at each amino acid position, and the median of the group mutation frequencies at each amino acid position was calculated from the individual means. Control subjects (A; n = 10), aviremic patients (B; n = 6), and viremic HIV-1-infected patients (C; n = 15).

The frequency of amino acid replacements by numbered position within the V_H_ molecule was identical in all groups (Figure 5), as was the proportion of non-conservative replacements at each site. The highest mutation frequencies were found in the CDR1/2 regions as were non-conservative mutations compared with those in FR1/2/3, suggesting antigen-driven selection requiring functional differences in structure compared with germline [24].

Binding of HIV-1 gp120 to V_H_ genes in a superantigen-like fashion has been reported to involve amino acid residues in FR1 (residues 10, 13, 19, 23, 28), CDR1 (residue 32), CDR2 (residues 54, 59, 64, 65), and FR3 (residues 75, 79, 81, 82a, 83, and 85) [26]. Despite suggestions that gp120 binding would affect SHM at these residues [26], both amino acid replacement frequencies and non-conservative-to-conservative ratios did not vary by group (Figure 5). Only amino acid 54 was less likely to have a non-conservative mutation in viremic patients compared with controls (p = 0.03).

### Nucleotide Mutation Patterns

Mutations in G and C are a direct result of the activity of activation-induced cytidine deaminase (AID). AID targets SHM mutations to “hotspots” (RGYW and complementary WRCY nucleotide motifs) [24],[27] that comprise a minority of total nucleotides but include the majority of mutations in CDR1/2 (Table 3). The numbers of these variable region motifs were similar (11–24 per sequence), and 59–61% of CDR mutations occurred in these hotspots, as did over a third of FR1/2/3 mutations in all groups.

**Table 3:** RGYW/WRCY motifs and targeted mutation frequencies in CDR and FR regions.

	Control	Aviremic	Viremic	p value
**Number of RGYW/WRCY motifs per V_H_ segment**	17.8 (11–23)	18.3 (13–23)	17.8 (13–24)	0.11
**% of CDR nucleotides mutated**	13.5 (7.4–15.2)	12.4 (10.2–14.1)	9.8 (4.9–14.4)	0.04^a^
**% of CDR mutations present in RGYW/WRCY motifs**	58.8 (48.5–62.5)	61.1 (56.8–71.0)	59.4 (53.5–69.2)	0.32
**% of FR nucleotides mutated**	5.2 (4.0–6.5)	4.8 (4.2–5.7)	4.1 (2.4–7.8)	0.18
**% of FR mutations present in RGYW/WRCY motifs**	29.3 (19.6–33.5)	30.1 (27.3–33.0)	28.7 (24.4–35.2)	0.55
**% of all nucleotides mutated**	7.2 (6.0–8.4)	6.5 (5.6–7.6)	5.3 (3.1–9.3)	0.14
**% of all mutations present in RGYW/WRCY motifs**	41.2 (31.9–46.3)	43.3 (39.9–48.6)	41.4 (37.1–45.6)	0.29

Group medians are listed with the range of individual means in each group in parentheses. Overall p values are listed on the table.

^a^ Secondary analyses: control vs. viremic p<0.05; all other comparisons p>0.05.

The initial deamination of cytidine nucleotides by AID [28] is subsequently repaired by translesion DNA synthesis (TLS) polymerases (DNA POLQ, POLZ, POLI, POLH, and REV1) yielding mutations in A and T [29]. Impairment of one or more of these polymerases can affect the resulting nucleotide mutation pattern [30]–[31]. Whereas all nucleotides tended to show lower mutation, the frequencies of CDR1/2 mutations of both A and G, relative to their numbers in the germline sequence, were significantly lower in viremic patients compared with controls (Table 4). In FR1/2/3 regions, G nucleotide mutations were again significantly lower among viremic patients. However, the relative frequency of mutations did not differ (G >A>C>T in CDRs, G>C>A>T in FR) between any group. Nucleotide patterns and proportions in aviremic patients were not significantly different than those in the control group.

**Table 4:** Nucleotide mutation patterns and proportions in IgG V_H_3 mRNA sequences.

	Control	Aviremic	Viremic	p value
**CDR**				
**% C nucleotides mutated**	13.03 (9.1–16.8)	13.8 (10.2–16.0)	10.29 (6.9–19.0)	0.13
**% G nucleotides mutated**	15.06 (17.5–19.2)	14.0 (12.9–17.8)	11.04 (6.2–16.9)	0.03^a^
**% A nucleotides mutated**	13.12 (6.8–16.2)	12.1 (8.1–13.8)	9.69 (3.9–14.4)	0.05^b^
**% T nucleotides mutated**	9.96 (6.9–11.5)	9.1 (8.0–10.3)	7.64 (2.5–13.4)	0.08
**FR**				
**% C nucleotides mutated**	5.32 (3.9–7.4)	5.4 (4.4–6.4)	3.97 (2.2–7.5)	0.21
**% G nucleotides mutated**	5.38 (4.2–7.5)	5.1 (4.7–6.1)	4.20 (2.6–8.7)	0.09
**% A nucleotides mutated**	5.77 (4.8–6.8)	5.4 (4.6–6.6)	4.83 (3.2–9.1)	0.23
**% T nucleotides mutated**	3.43 (2.2–4.0)	3.4 (2.6–4.1)	2.72 (0.9–5.2)	0.37
**CDR**				
**% of mutations that were C nts**	19.06 (17.5–26.8)	20.4 (18.1–22.5)	20.08 (17.0–29.1)	0.73
**% of mutations that were G nts**	30.85 (26.9–50.2)	33.1 (29.3–38.1)	31.33 (27.0–38.3)	0.58
**% of mutations that were A nts**	30.36 (26.9–35.0)	28.5 (24.7–35.3)	29.28 (24.6–36.3)	0.61
**% of mutations that were T nts**	19.33 (11.8–21.7)	17.4 (14.3–20.3)	17.06 (12.0–22.5)	0.28
**FR**				
**% of mutations that were C nts**	27.87 (23.8–33.6)	27.2 (25.8–30.7)	26.95 (22.7–33.0)	0.50
**% of mutations that were G nts**	35.70 (29.5–48.9)	35.0 (33.2–36.4)	34.14 (31.5–39.7)	0.32
**% of mutations that were A nts**	24.74 (21.7–35.0)	23.0 (20.9–24.6)	24.66 (21.2–29.4)	0.10
**% of mutations that were T nts**	13.32 (10.5–20.0)	14.4 (11.9–17.0)	14.36 (11.0–17.0)	0.59

“% C nucleotides mutated” indicates the proportion of nucleotides in either the CDR1/2 or FR1/2/3 regions that were mutated relative to the total number of C nucleotides present in the unmutated reference sequence, expressed as a percent. “% of mutations that were C nts” indicates the proportion of mutations in either the CDR1/2 or FR1/2/3 regions that were C nucleotides in the unmutated reference sequence relative to the total number of mutations in the region expressed as a percent. The group medians are listed (individual patient mean ranges in parentheses). Overall p values are listed on the table.

^a^ Secondary analyses: all comparisons p>0.05.

^b^ Secondary analyses: control vs. viremic p<0.05; all other comparisons p>0.05.

**Figure 6:**
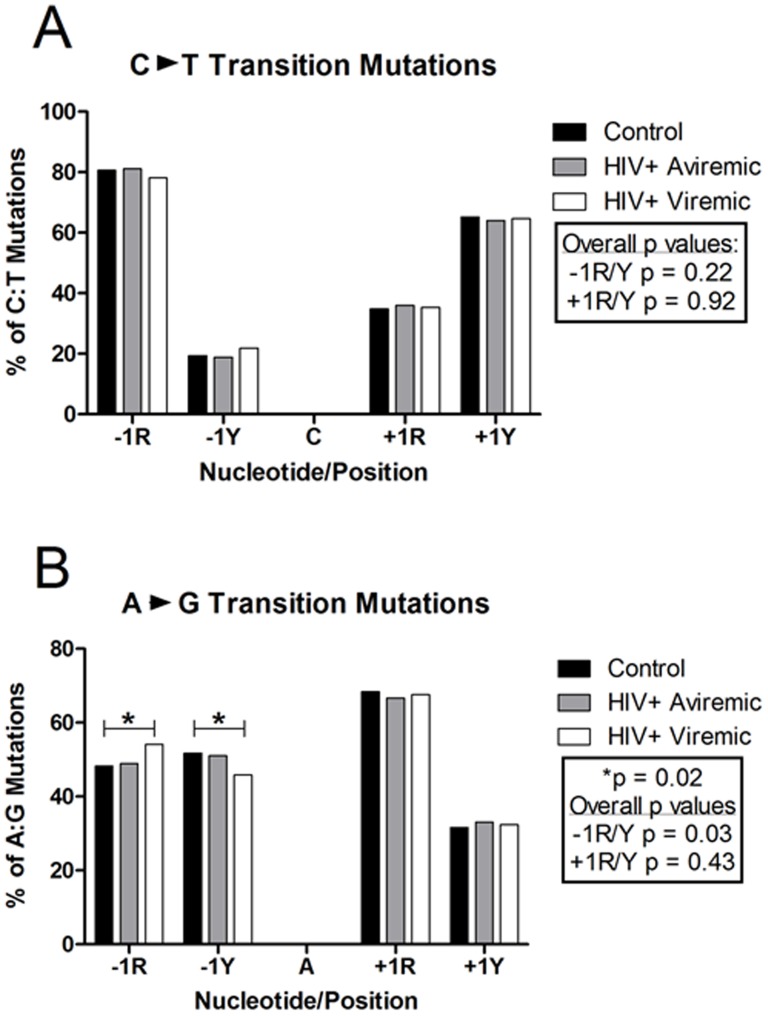
Dinucleotide analysis of C∶T and A∶G Transition mutations. The percent either of R (A or G) or Y (C or T) nucleotides occurring in either the −1 (5′) position or the +1 (3′) position to a mutation were calculated. Medians are represented for the control (n = 10; black bars), aviremic (n = 6; gray bars), and viremic HIV-1-infected groups (n = 15; white bars).

Characterization of neighboring bases can also be useful in examining both stages of SHM; AID-induction and subsequent TLS DNA polymerase-mediated repair. Consistent with previous data that AID-mediated mutation of cytidine nucleotides is determined in part by neighboring bases, particularly A or G in the +1 (3′) position [32], we showed that 76–82% of mutations of C occurred in the presence of an A or G in the adjacent +1 position (+1R, Figure 6a), regardless of whether the mutation resulted in a transition (C∶T) or a transversion (C∶A or C∶G) (Table S1). C or T were the preferred −1 (5′) adjacent nucleotides in 54–74% of C mutations (−1Y). These values are identical in each group. Mutations of G showed a very similar preference of −1 and +1 adjacent nucleotides [33]. Mutations of A and T were more variable in the preference of adjacent nucleotides depending on the type of resulting mutations. For A∶G transition mutations, an A or G in the −1 adjacent nucleotide was preferred (66–68%), whereas in A∶T transversion mutations a preference for C or T was seen. A∶C transversion mutations showed no significant preference at either the −1 or +1 position. The only difference seen between control subjects and viremic patients was in the preference at the −1 position in A∶G transition mutations. In controls, a −1 C or T was slightly preferred over A or G and in viremic patients a −1 A or G was slightly preferred over a C or T (Figure 6b). No differences were seen in the aviremic group compared with controls. C or T was the preferred nucleotide in both the −1 and +1 position for A∶T transversions (Table S1). An A or G in the −1 position of T mutations resulting in a transition or a transversion was found in 67–82% of mutations. However, only in the case of T∶A transversions was there any preference at the +1 position (A or G, 61–70%). These results, consistent with those in the literature [27] and now shown for the first time among patients with HIV-1 viremia, reveal that the process, if not the frequency of SHM, as well as SHM-associated lesion repair are intact in this group.

Finally, the preference of AID-induced mutations to be transitions (purine∶purine or pyrimidine∶pyrimidine) rather than transversions (purine∶pyrimidine) [24] is preserved in all groups (53–56% vs. 45–48%, respectively; data not shown). Overall, we show that the frequency of SHM is significantly decreased during viremic HIV-1 infection, particularly in the critical antigen-binding CDR regions. In contrast, the hierarchies in SHM and AID activity described in other non-HIV-1 groups [24],[27]–[31], such as nucleotide mutation patterns, mutation proportions, and mutation preferences, are maintained among patients with HIV-1 viremia.

## Discussion

We demonstrate on a molecular level that the frequency of SHM among circulating class-switched V_H_3-IgG B cells is decreased in patients with advanced viremic HIV-1-infection. SHM underlies the ability of B cells to make antibodies of high specificity, avidity and function. We targeted the V_H_3 genes due to their frequency (22 of 44 expressed V_H_ genes) and because V_H_3 antibodies are prominent in defense against invasive HIV-1-associated mucosally-acquired systemic pathogens [11]–[13] (e.g., *S. pneumoniae*, *H. influenzae*, and *Cryptococcus neoformans*) and in responses to associated preventive vaccines [11]–[13],[34]. Thus, a decreased ability to support SHM and, by inference, an impaired capacity to generate high affinity antigen-specific antibodies [6],[35]–[36] may underlie, in part, the high rates of invasive and often fatal HIV-1-associated bacterial infections [11],[37]–[39].

Three processes underlie the development of antibody diversity, specificity and function. The first, establishment of the primary B cell repertoire by V(D)J recombination, appears to be intact in HIV-1-infected patients. We found slightly decreased expression of only 3 of 22 V_H_3 genes in HIV-1-infected patients compared with controls, consistent with earlier data that the primary IgM^+^IgD^+^ repertoire in HIV-1-infected patients is intact [6]. Of note, selected V_H_3 genes (V_H_3-15, -30, 30.5, -73, and the prominent V_H_3-23) are reported to bind gp120 in a non-classical superantigen-like manner outside of the antigen-binding pocket [26]. In the absence of adequate T cell signals, such binding and activation could deplete these cells [40]. We identified only a decrement in V_H_3-23 expression, although lower numbers of sequences of the other genes may preclude finding such a difference. We also did not confirm suggestions that such putative gp120-binding residues would be more frequently mutated during SHM in expressed V_H_3^+^ B cells, nor in V_H_3^+^ B cells thought to be targets of gp120-binding [26]. Therefore, our data do not support preferential deletion of specific V_H_3 family members by gp120 binding nor preferential mutation at putative gp120 binding sites in the V_H_3 B cell repertoire. The few differences found in D and J_H_ gene expression may be reflective of exposure and infection by different pathogens than those that occur in controls, especially opportunistic pathogens which are well characterized during HIV-1 infection. Differences in D and J_H_ gene expression could also be due to the differential use of V_H_3 genes if certain V-D-J combinations are more common than others. However, differences in D and J_H_ regions, which make up the majority of the CDR3 region sequence, were not enough to result in any significant CDR3 region characteristics in either HIV-1-infected group. Therefore, we cannot predict the biological relevance of the differences in D and J_H_ regions.

The second and third processes, SHM and CSR, are both mediated by AID. We found no evidence of impaired CSR in serum *in vivo* or in culture *ex vivo* (e.g. IgG>IgA>IgM) in each group. Differences in the ratios of the isotypes present in blood compared with those produced *in vitro* may relate to the differing half lives of each antibody *in vivo* (IgG–21 days, IgA–10 days, IgM–6 days) and that most serum antibodies are produced in the marrow, not by circulating cells. However, for SHM, the frequencies of nucleotide and amino acid mutations were decreased, especially in the hypervariable antigen-binding CDR1/2 regions, of the V_H_3-IgG heavy chains from the HIV-1-infected patients. That the antigen-independent selection and frequencies of V(D)J genes were relatively similar in both groups suggests that the initial V(D)J recombination process is also relatively intact during HIV-1 infection. However, the defects in SHM occur with antigen stimulation and selection, processes that occur in germinal centers.

Indeed, germinal centers are smaller in patients such as ours with advanced HIV-1 infection, among whom the follicular dendritic cell (FDC) networks are attenuated and disrupted [41]. Such changes may limit the rigor of selection and serial mutation of antigen-specific B cells in HIV-1-infected patients. Alternatively, polyclonal bystander activation of non-specific and low affinity B cells by cytokines and growth factors (e.g. IFN-a, TNF-a, IL-4, IL-10, BAFF) and surface CD40L [42]–[43], which may be over-expressed in untreated HIV-1 infection [42], may also affect the selection process. Such non-specifically activated B cells may compete with antigen-specific activated B cells for survival factors [42], avoid deletion in attenuated FDC networks during the selection process and survive to migrate from germinal centers to the periphery. However, that the ratios of replacement/silent nucleotide mutations and of non-conservative/conservative amino acid mutations, both measures of selection, and similar in HIV-1-infected patients and controls, suggests that the process, if not the rigor, of selection may be relatively intact.

AID initiates SHM by deaminating cytidines, creating a U∶G lesion. The lesion can either be replicated over, creating a dC to dT transition mutation, or repaired by DNA repair pathways utilizing low fidelity translesion (TLS) DNA polymerases, creating additional mutations at A and T [31],[44]. Among CDR1/2 nucleotide studies herein, the significant decrease in overall mutation frequency, particularly in the percent of A and G mutations, does not match any phenotypes in cells or mice observed with selectively-deleted DNA repair pathways [31],[44]. Thus, our data do not support disruption of lesion repair and no effects of HIV-1 virions or proteins on DNA repair pathways are reported.

The lower frequencies of SHM but very similar patterns of mutation during HIV-1 viremia were instructive and surprising. Three studies show increased expression of AID, the enzyme that mediates SHM, among comparable patients with HIV-1 infection [45]–[47]. These findings would predict higher, not lower, mutation frequencies. Our data are consistent with results addressing mutation frequency in a small number of IgD^−^CD27^+^ memory B cells (which include IgG^+^ cells) that the mutation frequencies are lower than those in controls [47]. That AID mRNA expression was higher among CD27^+^ B cells is compatible with a murine study showing that high, constitutive AID expression was accompanied by decreased SHM, presumably due to negative regulation of AID activity [48]. In addition to influencing AID expression, HIV-1 can also have a negative effect on AID function. The HIV-1 protein Vif can block the SHM-associated activity of AID in *E. coli in vitro* by direct protein-protein interactions [49], although any effects of Vif-AID interactions on CSR or Vif activity *in vivo* are unknown. Similarly, the HIV-1 protein Nef has been shown to be taken up by B cells *in vivo* and interrupt CSR *in vitro* by blocking B cell activation pathways [50]. However, effects of Nef on SHM have not been determined.

Potential limitations of the study include the unequal distribution of age and gender among groups. Both HIV-1-infected groups, although older than controls, are distributed across the same age range. A model of our results that controlled for age showed a decline in mutation frequency with increasing age in these adults with HIV-1 infection. However, in contrast, four other reports highlight that V_H_ mutation frequency increases, rather than decreases with increasing age among persons without HIV-1 infection [51]–[54]. These findings suggest that the decreased mutation frequencies in patients with viremic HIV-1 infection are most likely confounded by HIV-1 status and viremia, rather than age. Indeed, in the literature, V_H_ gene mutation frequencies are either similar [51] or most often higher [52]–[54] in older adults compared with children and younger adults. Based on the literature cited, under the null hypothesis, our analysis assumes that the mutation frequency among our older age distribution would be higher rather than lower than that in younger adults, a result in the opposite direction of our results. That is, with the older age distribution in the HIV-1-infected adults, the ability of this infection to decrease V_H_ mutation frequencies would be dampened, and therefore, more difficult to detect. In addition, the median age was highest in the aviremic group. Despite this, mutation frequency in the aviremic group more closely resembled the control group rather than the viremic group, suggesting that HIV-1 viremia affects the SHM process.

Regarding gender we found no differences in amino acid mutation frequencies in CDR1/2 between male and female control subjects (25.8% (95% Confidence Interval C.I. = 18.1–29.7%) vs. 22.1% (C.I. = 15.2–31.0%), respectively, p = 0.61), again, in contrast to the lower mutation frequency among the predominantly male viremic patients and the higher mutation frequency among the control subjects with more females. Similarly, the aviremic population, composed of all males and with a similar ethnic background to the viremic group, had mutation frequencies that were a closer match to the control group than to the viremic group. Taken together with the age analysis above, these results support our conclusion that age, gender, and ethnic differences are not significantly confounding our results in this data set.

Due to the number of cells acquired from each patient, we sequenced expressed mRNA transcripts from circulating IgG^+^ class-switched B cells rather than sorted memory or plasma cells. Class switching to an IgG isotype is unlikely but possible in B cells outside of a germinal center environment. In addition, although multiple identical sequences were isolated from patients, only one of these sequences was included from each patient to prevent overrepresentation of a single clone or plasmablast. In the patients among whom flow cytometry was performed, we identified no significant difference in the proportion of CD19^+^CD27^+^ memory B cells in the HIV-1-infected patients compared with control subjects (control: n = 5, median 18.5%, range 12.2–39.2%; aviremic: n = 6, median 17.0%, range 7.3–27.1%; viremic: n = 7, median 22.3%, range 6.5–25.2%; overall p = 0.80). Therefore, we assume that the B cells sampled are all single IgG^+^ circulating memory or plasmablast cells that have matured in the context of a germinal center reaction. Future experiments are being directed to identify specific B cell subtypes that may harbor the lower mutation frequencies in HIV-1-infected patients.

In summary, we report a significant decrement in SHM in V_H_3-IgG mRNA transcripts from patients with advanced, viremic HIV-1 infection compared with those from control subjects. Such deficits may involve decreases in the activity of AID, which can occur by several mechanisms that may change the context in which AID activation occurs, or selection in germinal centers. These results may underlie, in part, the poor quality of vaccine responses and the high incidences of invasive bacterial infections in patients with untreated HIV-1 viremia. Uncovering the mechanism underlying the decreased SHM frequencies may facilitate development of interventions to enhance the quality of antibody responses to infection and vaccination in patients with HIV-1 infection in the U.S. and in resource-limited settings, where the incidence and impact of these serious infections is greatest.

## Supporting Information

Table S1 **Nucleotide mutations and adjacent nucleotide patterns in IgG V_H_3 mRNA sequences.** The ratios of purine (R; A or G) and pyrimidine (Y; C or T) nucleotides adjacent to mutations are listed in table S1 for both the −1 position (nucleotide preceding the mutation) and the +1 position (nucleotide succeeding the mutation).
(DOCX)
