# Supplementary material for: Decreased Mutation Frequencies among Immunoglobulin G Variable Region Genes during Viremic HIV-1 Infection
Source: PLoS One. 2014 Jan 7;9(1):e81913. doi: 10.1371/journal.pone.0081913 (PMC3883639; doi:10.1371/journal.pone.0081913)
Supplement: Table S1 — Nucleotide mutations and adjacent nucleotide patterns in IgG VH3 mRNA sequences. The ratios of purine (R; A or G) and pyrimidine (Y; C or T) nucleotides adjacent to mutations are listed in table S1 for both the −1 position (nucleotide preceding the mutation) and the +1 position (nucleotide succeeding the mutation). (DOCX) [file pone.0081913.s001.docx]

**Supplemental Table S1. Nucleotide mutations and adjacent nucleotide patterns in IgG V_H_3 mRNA sequences.**

| Mutation | R:Y Ratio |  |  | Mutation | | | R:Y Ratio | |  |
| --- | --- | --- | --- | --- | --- | --- | --- | --- | --- |
|  | **5’** | **3’** |  | |  | **5’** | | **3’** | |
| **C → G (Tr)** |  |  |  | | **G → C (Tr)** |  | |  | |
| Control | 4.9 : 1 | 1 : 2.2 |  | | Control | 2.7 : 1 | | 1 : 2.5 | |
| Aviremic | 3.9 : 1 | 1 : 2.6 |  | | Aviremic | 3.0 : 1 | | 1 : 2.4 | |
| Viremic | 4.2 : 1 | 1 : 2.9 |  | | Viremic | 2.9 : 1 | | 1 : 2.4 | |
| p value | 0.47 | 0.45 |  | | p value | 0.93 | | 0.98 | |
| **C → A (Tr)** |  |  |  | | **G → A (Ts)** |  | |  | |
| Control | 3.2 : 1 | 1 : 1.2 |  | | Control | 2.1 : 1 | | 1 : 3.1 | |
| Aviremic | 3.1 : 1 | 1 : 1.3 |  | | Aviremic | 2.1 : 1 | | 1 : 2.8 | |
| Viremic | 4.0 : 1 | 1 : 1.3 |  | | Viremic | 2.1 : 1 | | 1 : 2.9 | |
| p value | 0.37 | 0.60 |  | | p value | 0.74 | | 0.30 | |
| **C → T (Ts)** |  |  |  | | **G → T (Tr)** |  | |  | |
| Control | 4.2 : 1 | 1 : 1.9 |  | | Control | 1 : 1.1 | | 1 : 3.1 | |
| Aviremic | 4.3 : 1 | 1 : 1.8 |  | | Aviremic | 1 : 1.0 | | 1 : 3.3 | |
| Viremic | 3.6 : 1 | 1 : 1.8 |  | | Viremic | 1.2 : 1 | | 1 : 3.6 | |
| p value | 0.28 | 0.80 |  | | p value | 0.42 | | 0.09 | |
|  |  |  |  | |  |  | |  | |
| **A → C (Tr)** |  |  |  | | **T → C (Ts)** |  | |  | |
| Control | 1 : 1.1 | 1.3 : 1 |  | | Control | 1.6 : 1 | | 2.7 : 1 | |
| Aviremic | 1 : 1.2 | 1.1 : 1 |  | | Aviremic | 1.5 : 1 | | 2.4 : 1 | |
| Viremic | 1 : 1.1 | 1.4 : 1 |  | | Viremic | 1.5 : 1 | | 2.7 : 1 | |
| p value | 0.85 | 0.85 |  | | p value | 0.10 | | 0.76 | |
| **A → G (Ts)** |  |  |  | | **T → G (Tr)** |  | |  | |
| Control | 1 : 1.1 | 2.2 : 1 |  | | Control | 1 : 1.2 | | 2.3 : 1 | |
| Aviremic | 1 : 1.0 | 2.0 : 1 |  | | Aviremic | 1 : 1.4 | | 2.6 : 1 | |
| Viremic | 1.2 : 1 | 2.1 : 1 |  | | Viremic | 1 : 1.1 | | 2.1 : 1 | |
| p value | 0.02 | 0.30 |  | | p value | 0.52 | | 0.93 | |
| **A → T (Tr)** |  |  |  | | **T → A (Tr)** |  | |  | |
| Control | 1 : 2.8 | 1 : 2.2 |  | | Control | 1.6 : 1 | | 4.8 : 1 | |
| Aviremic | 1 : 3.6 | 1 : 2.1 |  | | Aviremic | 1.7 : 1 | | 4.5 : 1 | |
| Viremic | 1 : 3.6 | 1 : 2.1 |  | | Viremic | 2.4 : 1 | | 3.5 : 1 | |
| p value | 0.13 | 0.44 |  | | p value | 0.17 | | 0.23 | |

R = A or G; Y = C or T. Mutations: Ts = transition (purine ↔ purine or pyrimidine ↔ pyrimidine); Tr = transversion (purine ↔ pyrimidine).
